# Complete chloroplast genome features and phylogenetic analysis of *Abies ernestii* var. *salouenensis* (Bordères and Gaussen) W. C. Cheng and L. K. Fu from southwest China

**DOI:** 10.1080/23802359.2023.2209384

**Published:** 2023-05-12

**Authors:** Yi-Zhen Shao, Zhao Wang, Wen-Jun Liu, Peng-Fei Zhao, Si Wu

**Affiliations:** a College of Life Sciences, Henan Agriculture University, Zhengzhou, China; b School of Life Sciences, Zhengzhou Normal University, Zhengzhou, China

**Keywords:** *Abies ernestii* var. *salouenensis*, comparative genome analyses, complete chloroplast genome, phylogenetic analysis

## Abstract

*Abies ernestii* var. *salouenensis* (Bordères & Gaussen) W. C. Cheng & L. K. Fu is endemic to southwest China, including the southeastern Tibetan Plateau and the northwestern Yunnan Province. The taxonomic relationships between *A. ernestii var. salouenensis* and two other closely related fir species (*A. chensiensis* Tiegh. and *A. ernestii* Rehd.) still need to be determined. Here, we report for the first time the whole chloroplast genome of *A. ernestii* var. *salouenensis*. Its genome is 121,759 bp long and is characterized by a circular structure with 68 peptide-encoding genes, 16 tRNAs, six ORFs, and four rRNAs. We also identified 70 microsatellite repeat sequences and 14 tandem repeat sequences in the chloroplast genome of *A. ernestii* var. *salouenensis*. Comparative genome analysis indicated considerable variation in *ycf*1 and *ycf*2. Phylogenetic analysis supported the monophyly of *A. ernestii* var. *salouenensis*, *A. chensiensis* Tiegh., and *A. ernestii* Rehd. The relationships among them should be surveyed using more samples at the species level. This study will facilitate taxonomic studies and the development of suitable chloroplast markers for fir species.

## Introduction

**Figure 1.**
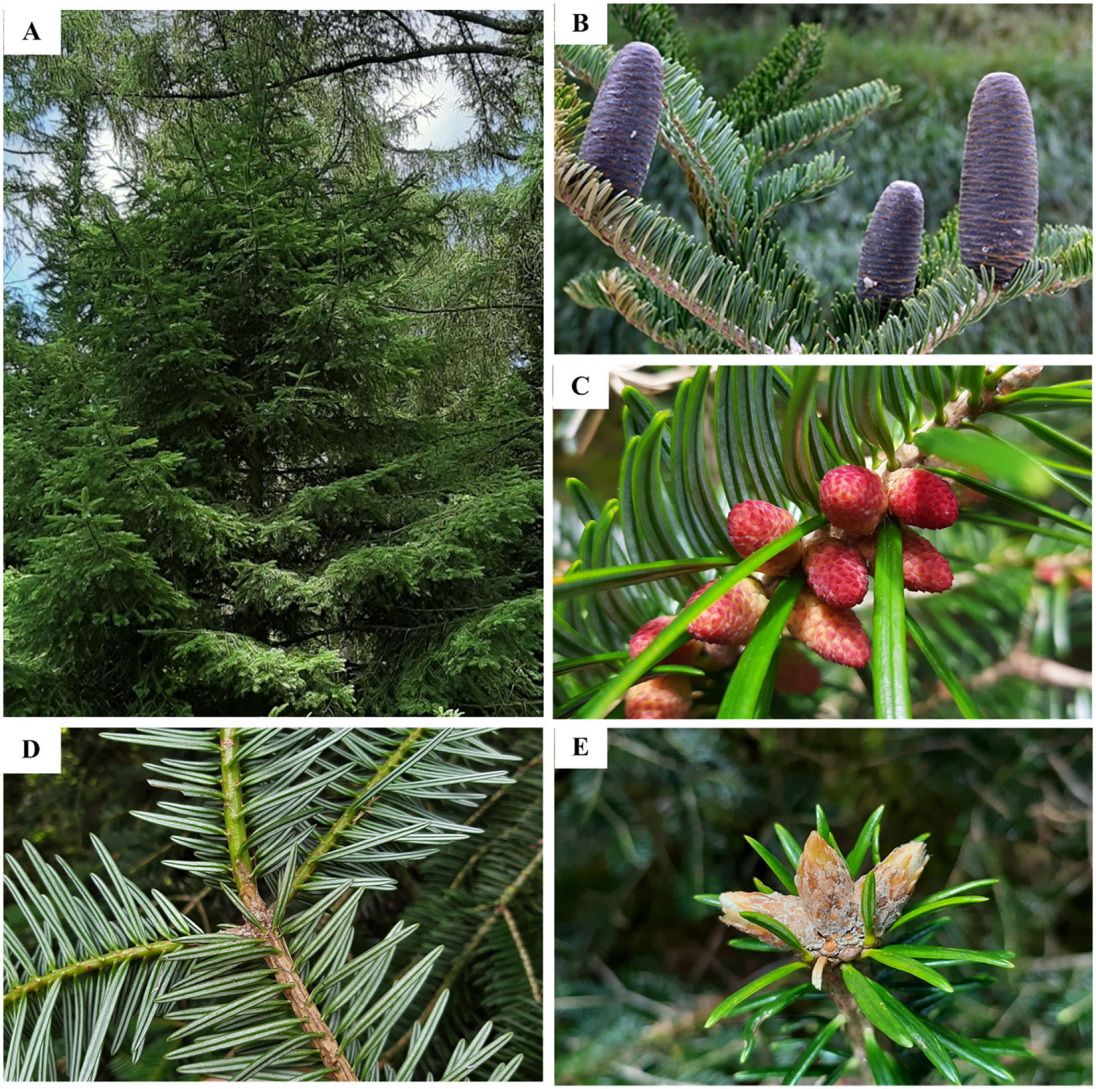
*Abies ernestii* var. *salouenensis*. (A) Plant; (B) mature cones; (C) pollen cones; (D) leaves; (E) vegetative buds. M. These images are from Christian (2021) and photography by Yi-Zhen Shao.

*Abies ernestii* var. *salouenensis* (Bordères & Gaussen) W. C. Cheng and L. K. Fu is endemic to southwest China, including the northwest Yunnan Province and the southeastern Tibetan Plateau (Figure 1) (Kuan 1981; Farjon 1990). It serves as an essential habitat for many plants and animals, making it an ecologically significant component of the cold-temperate woods (Farjon and Rushforth 1989). To date, the complete chloroplast genome features of *Abies ernestii* var. *salouenensis* has never been investigated.

Species delimitation of *A. ernestii* var. *salouenensis* and two other closely related species (*A. chensiensis* Tiegh. and *A. ernestii* Rehd.) has been a contentious issue for a long time. (Suyama et al. 2000; Xiang et al. 2009; Semerikova et al. 2011, 2018; Shao and Xiang 2015). Unlike other fir species, these three closely related fir species have particular habitat feature such as lower altitude ranges, relatively arid habitat, and neutral to slightly alkaline soils (Liu 1971; Farjon 2001). A series of studies using morphological characteristics and molecular markers have been conducted to address this taxonomic problem (Xiang et al. 2004, 2015). Unfortunately, the three closely related fir species have never been simultaneously surveyed using the recommended high-resolution markers (e.g. chloroplast genomes) (Liepelt et al. 2010; Aguirre-Planter et al. 2012; Xiang et al. 2009, 2018). Consequently, several distinct taxonomic pairings have been proposed: *Abies ernestii* var. *salouenensis* was once thought to be a subspecies or variety of *A. chensiensis* (Rushforth 1984; Shao and Xiang 2015); and *A. ernestii* var. *salouenensis*, *A. ernestii*, and *A. chensiensis* were treated identically by Handel-Mazzetti (1929) and Dallimore and Jackson (1966). Recently, complete chloroplast genome data have emerged as one of the most effective indicators for distinguishing taxonomically complex groups (Shao et al. 2022). Thus, a more thorough approach utilizing chloroplast genomes is required to elucidate the relationships among these three closely related fir species.

In this study, we constructed and sequenced the chloroplast genome of *A. ernestii* var. *salouenensis*, and then conducted a comparative analysis with *A. chensiensis* and *A. ernestii*. This will facilitate taxonomic studies and the development of suitable chloroplast markers for fir species.

## Materials and methods

### Plant collection and DNA extraction

The leaves of *A. ernestii* var. *salouenensis* were collected by Qiao-Ping Xiang from Deqen County, Yunnan Province, China (E98.90°, N28.18°). A voucher specimen (Voucher Number: BMXDC) was preserved in the herbarium of the Institute of Botany, CAS (PE) (http://pe.ibcas.ac.cn, Qin Ban, herbarium2@ibcas.ac.cn). The entire genome of *A. ernestii* var. *salouenensis* was extracted using an EZNA Plant DNA Extraction Kit (OMEGA, USA).

### Genome sequencing, assembly, and annotation

A genomic library was constructed using the TruSeq Nano DNA Sample Prep Kit (Illumina, USA) according to the manufacturer’s protocol. The libraries were 150 bp long and sequenced on an Illumina HiSeq X platform. GeSeq, tRNAscan-SE v1.3.1, and a CLC de novo assembler (CLC Bio, Aarhus, Denmark) were used for further alignment, assemble, and annotation of reads (Schattner et al. 2005; Tillich et al. 2017). The readings were edited using quality restriction of Q5 and *N* > 10% to ensure excellent quality. These reads were alligned to the reference sequence using Velvet (Zerbino and Birney 2008). To match the gene predictions, we checked all the start/stop codons and intron/exon boundaries in Sequin and Geneious (Kearse et al. 2012; Lohse et al. 2013). Finally, the sequences were annotated by comparing them with published genomes. The overall coverage depth of the chloroplast genome assembly of *Abies ernestii* var. *salouenensis* was 124× (Figure S1). The associated GenBank accession number was MH706708.

### Repeat sequences detection and comparative genomic analysis

The MISA program was used to examine simple sequence repeats (SSRs). The REPuter website was used to survey long repeats (Kurtz et al. 2001). The maximum and minimum computed repeats were 50 bp and 30 bp, respectively. The Hamming distance was set to three. Next, we examined the complete chloroplast genomes of *A. ernestii* var. *salouenensis* (MH706708), *A. ernestii* (MH706707), and *A. chensiensis* (MH047653 and MH706706), which was available in the NCBI Database. mVista was used for comparative genomic analysis in the Shuffle-LAGAN mode (Frazeret al. 2004). Ten repeating units for mono-nucleotides, five for di-nucleotides, four for tri-nucleotides, three for tetra-nucleotides, three for penta-nucleotides, and three for hexa-nucleotides were set as the appropriate repeat units.

### Phylogenetic analysis

Phylogenetic analysis was performed using 23 reported chloroplast genomes of fir species and *Keteleeria davidiana* (Bertr.) Beissn. as an outgroup. These 23 genomes represented the main clades of the genus *Abies*, and all are closely related species of *A. ernestii* var. *salouenensis* (Xiang et al. 2018). Whole genomes were aligned using MAFFT v.7 (Katoh and Standley 2013). We performed and visualized the maximum likelihood (ML) analysis (bootstrap search steps = 1000) using RAxML v.8.1 and FigTree v.1.4 (Bootstrap search steps = 1000), respectively (Stamatakis 2014). The details of the 23 complete chloroplast genomes are listed in Table S1.

## Results and discussion

### Chloroplast genome features of A. ernestii var. salouenensis

**Figure 2.**
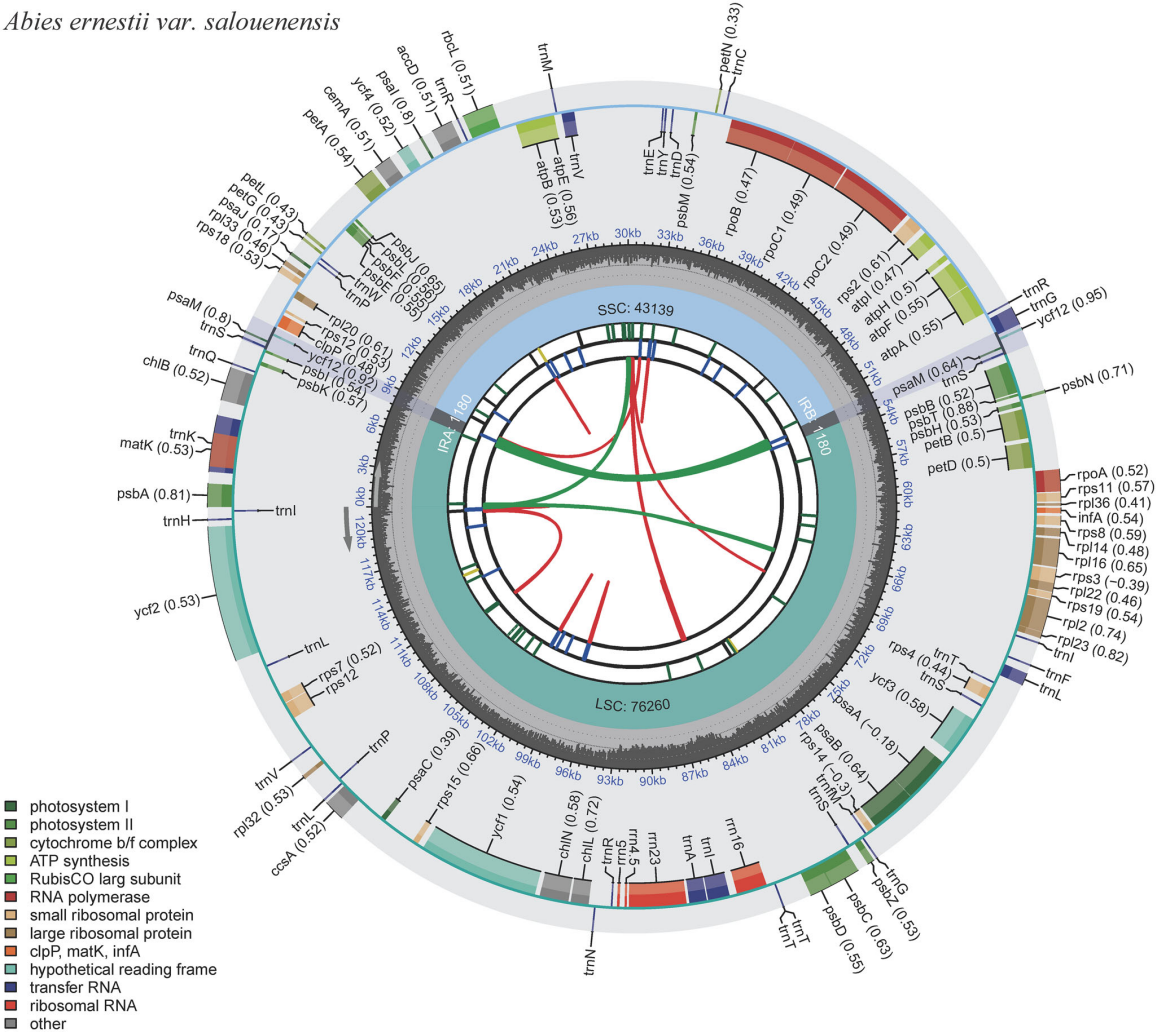
Schematic map of overall features of the chloroplast genome of *Abies ernestii* var. *salouenensis*. The circular map of the chloroplast genome was generated using CPGview (Liu et al. 2023). Genes shown outside the circle are transcribed clockwise, and genes inside are transcribed counter-clockwise. Genes belonging to different functional groups are color-coded. The darker gray in the inner corresponds to the GC content, and the lighter gray to the AT content.

The whole chloroplast genome of *A. ernestii* var. *salouenensis* displayed a circular DNA molecule of 121,759 bp in length. The genome displayed a significant A/T bias of 61.70%, similar to that of other fir species (Shao et al. 2022). It possessed a normal quadripartite structure with two IR regions (264 bp), one long single-copy region (LSC) region (67,155 bp), and one short single-copy region (SSC) region (54,076 bp). We identified 3 open reading frames (ORFs), 53 protein-coding genes, and 16 tRNA genes in the LSC. The SSC region was home to all four rRNAs. The IR region was 264 bp long and contained the *trnI*-*CAU* and *trnT*-*GGU* genes. We identified 113 genes, including 68 peptide-encoding genes (CDS), 16 transfer RNAs (tRNA), 6 ORFs, and 4 ribosomal RNAs (rRNA) (Table S2, Figure 2). The *clpP* and *ycf3* contained two introns, whereas only one intron occurred in *atpF*/*rps12*/*trnL*-*UAA*/*rpl16*/*petB*/*trnV*-*UAC*/*petD*/*trnA*-*UGC*/*rpoC1*/*trnG*-*GCC*/*trnK*-*UUU* (Table 1, Figure S2). Similar to other fir species, there was a palindromic inverted 1180 bp-repeat (*ycf12*/*trnS*/*psaM*/*trnG*) in the 52-kb inversion points, and the *ndh* genes were absent (Shao et al. 2022). The widespread absence of *ndh* genes in the Pinaceae can be explained by their high substitutability (Blazier et al. 2011).

**Table 1.** List of genes encoded in *Abies ernestii* var. *salouenensis* chloroplast genomes.

Groups of genes	Name of genes
Ribosomal RNAs	*rrn16, rrn23, rrn4.5, rrn5*
Transfer RNAs	trnA-UGC^a^, trnC-GCA, trnD-GUC, trnE-UUC, trnF-GAA, trnfM-CAU, trnG-GCC^a^, trnG-UCC , trnH-GUG, trnI-CAU*, trnI-GAU^a^, trnK-UUU^a^, trnL-CAA, trnL-UAA^a^, trnL-UAG, trnM-CAU, trnN-GUU, trnP-GGG, trnP-UGG, trnQ-UUG, trnR-ACG, trnR-CCG, trnR-UCU, trnS-GCU*, trnS-GGA, trnS-UGA, trnT-GGU*, trnT-UGU, trnV-GAC, trnV-UAC^a^, trnW-CCA, trnY-GUA
Subunits of photosystem I	psaA, psaB, psaC, psaI, psaJ, psaM*
Subunits of photosystem II	*psbA, psbB, psbC, psbD, psbE, psbF, psbH, psbI, psbJ, psbK, psbL, psbM, psbN, psbT, psbZ*
Subunits of cytochrome b/f complex	*petA, petB^a^, petD^a^, petG, petL, petN*
Subunits of ATP synthase	*atpA, atpB, atpE, atpF^a^, atpH, atpI*
Proteins of large ribosomal subunit	*rpl2^a^, rpl14, rpl16^a^, rpl20, rpl22, rpl23, rpl32, rpl33, rpl36*
Proteins of small ribosomal subunit	*rps2, rps3, rps4, rps7, rps8, rps11, rps12^b^, rps14, rps15, rps18, rps19*
Large subunit of RuBisco	*rbcL*
Subunits of RNA polymerase	*rpoA, rpoB, rpoC^a^, rpoC2*
Conserved hypothetical chloroplast reading frames	ycf1, ycf2, ycf3^b^, ycf4, ycf12*
ATP-dependent protease subunit P	*clpP*
Chloroplast envelope membrane protein	*cemA*
Chlorophyll biosynthesis	*chlB, chlL, chlN*
Miscellaneous proteins	*accD, ccsA, infA, matK*

*Genes with two copies; ^a^Genes with one intron. ^b^Genes with two introns.

### Repeat sequence analysis

Due to their excellent reliability, simple sequence repeats (SSRs) have been utilized extensively in phylogenetic research (Kaur et al. 2015). We identified 70 SSRs containing mononucleotide repeats (44), dinucleotide repeats (14), trinucleotide repeats (2), tetranucleotide repeats (8), and pentanucleotides repeats (2) in the chloroplast genome of *A. ernestii* var. *salouenensis* (Figure S3). Mononucleotide repeats (62.86%) were most abundant, followed by pentanucleotides (2.86%) and trinucleotide repeats (2.86%). This indicates that mononucleotide repeats contributed the most to genetic diversity. We identified two kinds of trinucleotide SSRs (ATT/AAT), twelve kinds of tetranucleotide SSRs (AAAT/ATCT/AAAG/AGAT/ATTT/ACCT/AACC/GGTT/ATCC/CTTT/ATGG/AGGT), and four kinds of pentanucleotide SSRs (ATTCG/ATGTT/AACAT/AATCG/) (Figure S3). In addition, 51 long repeats, including palindromic repeats (15), tandem repeats (14), and forward repeats (22), were identified (Figure S3).

### Comparative genome analysis

The IR region was significantly more conserved than the LSC and SSC sections in the comparative genome analysis. A significant bulk of the genetic diversity in these three fir species was contained in the noncoding and intergenic regions. This may explain the absence of DNA markers in closely related fir species in published research (Shao and Xiang 2015; Xiang et al. 2018; Shao et al. 2020). In addition, we compared the genomes of these three species to aid the search for applicable DNA barcodes (Figure S4). In our analysis, only *ycf1* and *ycf2* were characterized by considerable variation and could be suggested as potential chloroplast markers (Figure S4). As reported by Dong et al. (2015), *ycf1* is rich in short repeats and may be the most effective plastid barcode. In addition, the widely used markers in fir species (e.g. *rpl16, matK*, *trnC-D, rps18, trnS-G*) were lack of resolution among these three fir species (Shao et al. 2018).

### Phylogenomic analysis

**Figure 3.**
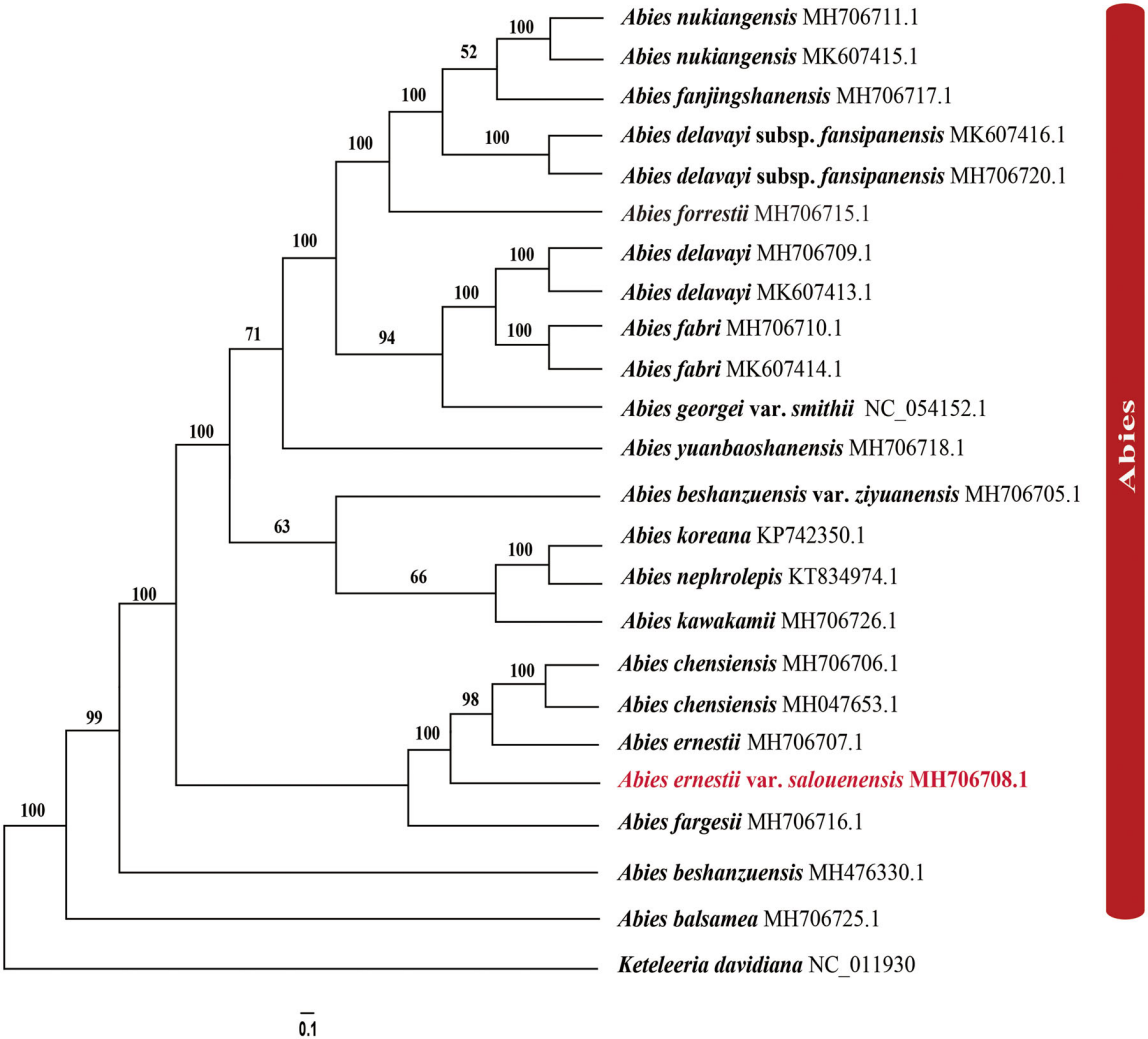
The best Maximum likelihood (ML) phylogram inferred from 23 chloroplast genomes in *Abies*, with *Keteleeria davidiana* as an outgroup (bootstrap values are indicated on the branches). The following sequences were used: *A. nukiangensis* Cheng et L. K. Fu MH706711 and MK607415 (Shao et al. 2020), *A. fanjingshanensis* W. L. Huang, Y. L. Tu and S. Z. Fang MH706717 (Guo et al. 2019), *A. delavayi* subsp. *fansipanensis* (Q.P.Xiang, L.K.Fu and Nan Li) Rushforth MH706720 and MK607416 (Shao et al. 2020), *A. forrestii* C. C. Rogers MH706715 (Dong et al. 2021), *A. delavayi* Franch. MH706709 and MK607413 (Shao et al. 2020), *A. fabri* (Mast.) Craib MH706710 and MK607414 (Shao et al. 2020), *A. georgei* var. *smithii* (Viguie et Gaussen) Cheng et L NC_054152 (Li et al. 2021), *A. yuanbaoshanensis* Y. J. Lu & L. K. Fu MH706718 (Zhang et al. 2020), *A. beshanzuensis var. ziyuanensis* (L. K. Fu & S. L. Mo) L. K. Fu & Nan Li MH706705 (Fu et al. 2019), *A. koreana* E. H. Wilson KP742350 (Yi et al. 2015), *A. nephrolepis* (Trautv.) Maxim. KT834974 (Yi et al. 2016), *A. kawakamii* (Hayata) T. Ito MH706726 (Shao et al. 2019), *A. chensiensis* MH706706 and MH047653 (Liu et al. 2018; Su et al. 2019), *A. ernestii* MH706707 (Shao et al. 2022), *A. fargesii* Franch. MH706716 (Guo et al. 2019), *A. beshanzuensis* M. H. Wu MH 476330 (Shao et al. 2018), *A. balsamea* (L.) Mill. MH 706725 (Wu et al. 2019), and *Keteleeria davidiana* NC_011930 (www.ncbi.nlm.nih.gov/).

The rapid development of new approaches has significantly expanded the available chloroplast genome data. To infer the phylogenetic relationships between *A. ernestii* var. *salouenensis*, *A. ernestii*, and *A. chensiensis*, we selected 23 reported chloroplast genomes of fir species, using *Keteleeria davidiana* as an outgroup (Figure 3). Phylogenetic analyses showed that *Abies* species formed a monophyletic lineage (BS_ML_ = 100). Within the genus *Abies*, the species from North America (*A. balsamea* (L.) Mill.) and East Asia formed a clear sister lineage (BS_ML_ = 100). In the East Asian clade, *Abies ernestii* var. *salouenensis*, *A. ernesti*, and *A. chensiensis* formed a monophyletic lineage (BS_ML_ = 100). Our results further indicated that *A. ernesti* was much more similar to *A. chensiensis* rather than its subspecies, *Abies ernestii* var. *salouenensis* (BS_ML_ = 96) (Figure 3). Therefore, the status of *A. ernestii* var. *salouenensis* as a variety of *A. ernestii* might not be supported. However, the phylogenetic relationships among the three species were polyphyletic (Figure 3). This could be explained by the limited sample size (*N* = 1 or 2) and the influence of hybridization (Xiang et al. 2015; Shao et al. 2020; Shao et al. 2022). Considering the above results, our phylogenomic analyses proved that chloroplast genome data are an effective indicator for distinguishing closely related fir species. Future studies should include additional individuals at the species level.

This study offers new evidence and validates the potential reliability of using complete chloroplast genomes for problematic firs. These findings indicate a vital genetic resource for ecologically significant fir species.

## Supplementary Material

Supplemental Material

Supplemental Material

Supplemental Material

Supplemental Material

Supplemental Material

## Data Availability

The genomic sequence data supporting the findings of this work are accessible through GenBank of NCBI at (https://www.ncbi.nlm.nih.gov/) with the accession number MH706708. The related BioProject, SRA, and Bio-Sample identifiers are PRJNA790665, SRP351584, and SAMN24219952.
