# Supplementary figures and images for: Complete chloroplast genome features and phylogenetic analysis of Abies ernestii var. salouenensis (Bordères and Gaussen) W. C. Cheng and L. K. Fu from southwest China
Source: Mitochondrial DNA B Resour. 2023 May 12;8(5):550–6. doi: 10.1080/23802359.2023.2209384 (PMC10184594; doi:10.1080/23802359.2023.2209384)

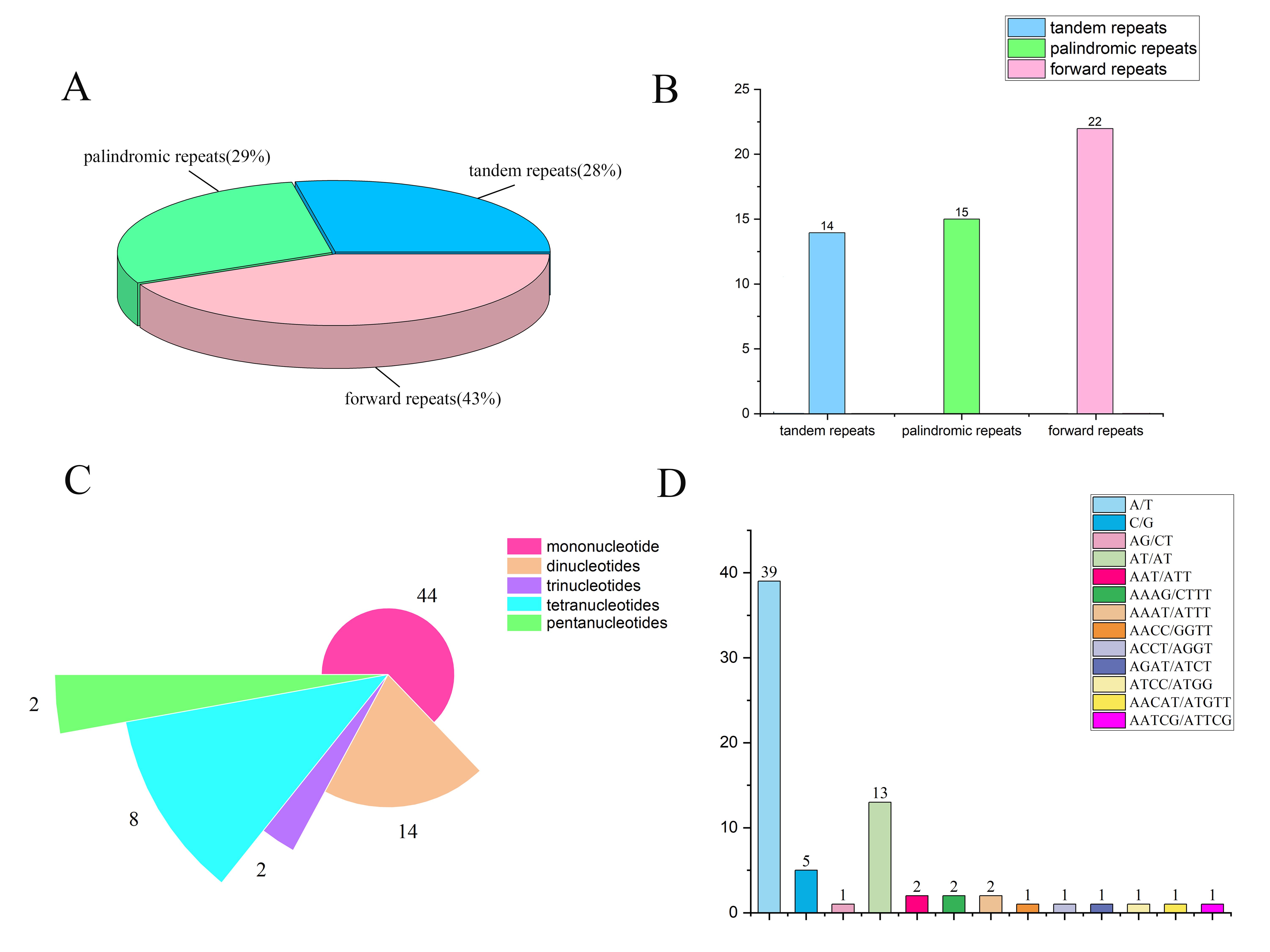

Supplement: Supplemental Material [file TMDN_A_2209384_SM1335.tif]

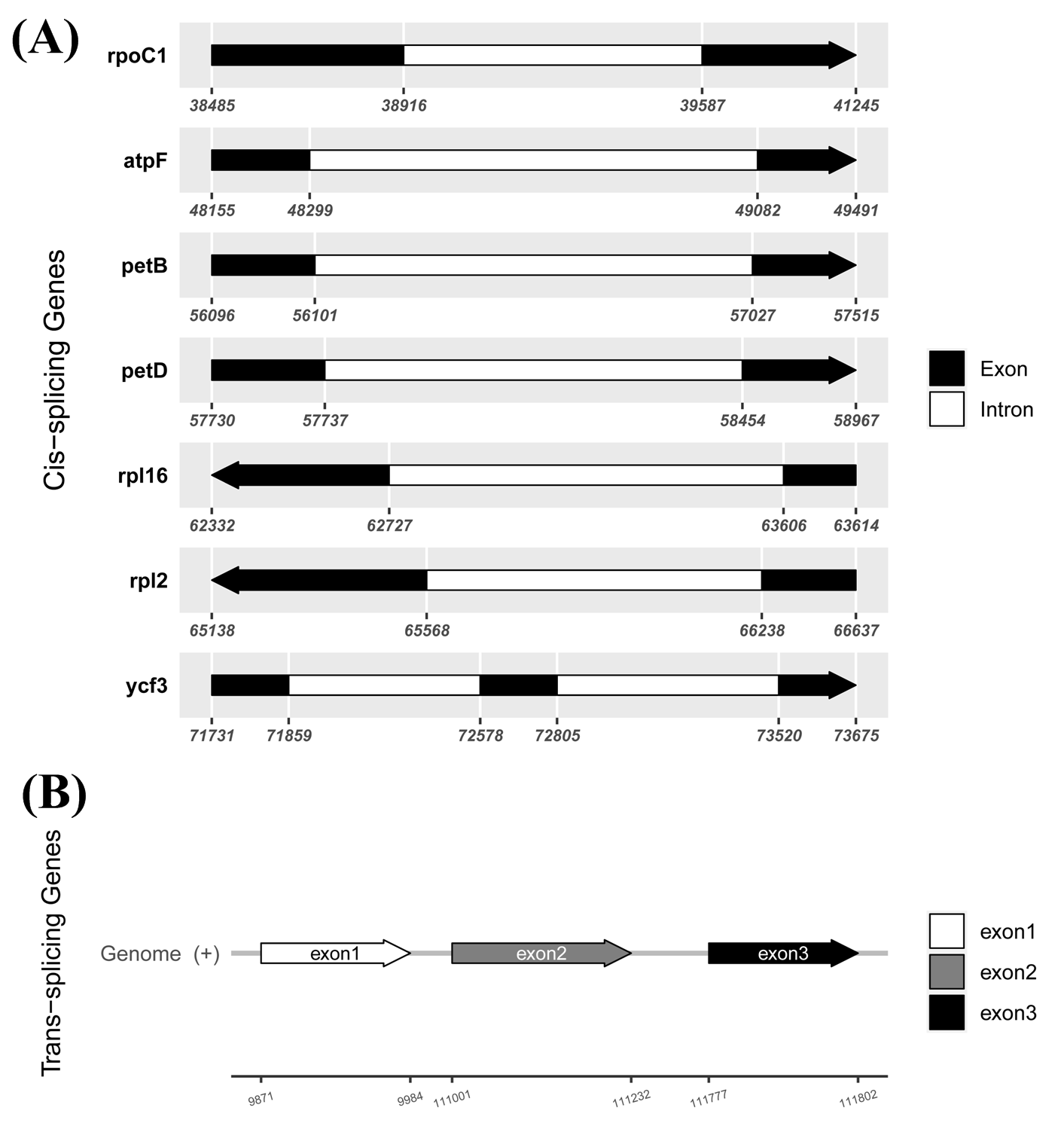

Supplement: Supplemental Material [file TMDN_A_2209384_SM1334.tif]

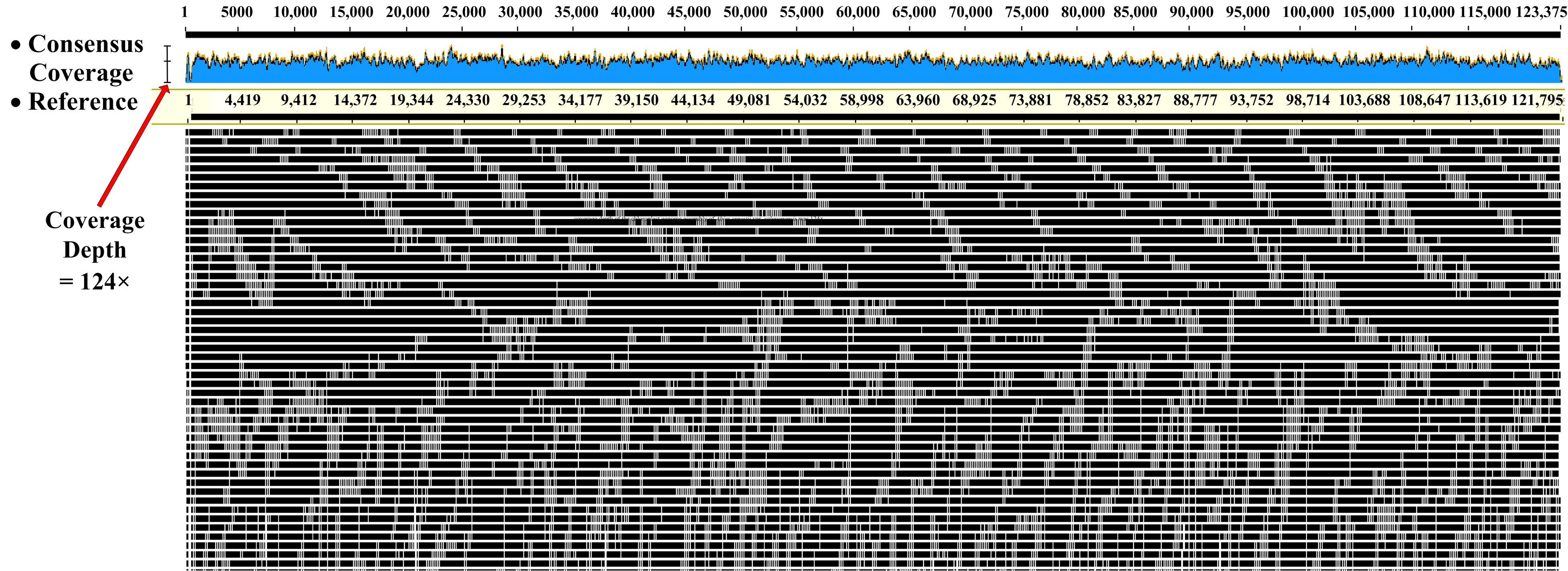

Supplement: Supplemental Material [file TMDN_A_2209384_SM1333.tif]
