# Supplementary material for: Complete chloroplast genome features and phylogenetic analysis of Abies ernestii var. salouenensis (Bordères and Gaussen) W. C. Cheng and L. K. Fu from southwest China
Source: Mitochondrial DNA B Resour. 2023 May 12;8(5):550–6. doi: 10.1080/23802359.2023.2209384 (PMC10184594; doi:10.1080/23802359.2023.2209384)
Supplement: Supplemental Material [file TMDN_A_2209384_SM1323.doc]

**Supplementary material**

The online material available for this article contains the following:

**Table S1.** The complete chloroplast genomes used in this study.

**Table S2.** The chloroplast genome organization of *Abies ernestii* var. *salouenensis*.

**Figure S1**. Overall coverage depth of the chloroplast genome assembly of *Abies ernestii* var. *salouenensis*.

**Figure S2**. Schematic map of the cis and trans splicing genes in the chloroplast genome of *Abies ernestii* var. *salouenensis*.

**Figure S3**. Types and amount of SSRs (A and B) and long sequence repeats (C and D) in the chloroplast genome of *Abies ernestii* var. *salouenensis*.

**Figure S4.** Comparison of four chloroplast genomes using the mVista alignment program, with *Abies chensiensis* (MH706706) as a reference. The X-axis means the window's midpoint, and the y-axis means nucleotide diversity (Pi). Genome regions are colour-coded as protein-coding, rRNA coding, tRNA coding, or conserved noncoding sequences.

**Table S1.** The complete chloroplast genomes used in this study.

| **Family** | **Genus** | **Species** | **GenBank accession number** | | **Length** |
| --- | --- | --- | --- | --- | --- |
| Pinaceae | *Abies* | *Abies balsamea* (L.) Mill. | | MH 706725 | 121,574 bp |
| Pinaceae | *Abies* | *Abies beshanzuensis* M. H. Wu | | MH 476330 | 121,399 bp |
| Pinaceae | *Abies* | *Abies beshanzuensis* var. *ziyuanensis* (L. K. Fu & S. L. Mo) L. K. Fu & Nan Li | | MH 706705 | 121,274 bp |
| Pinaceae | *Abies* | *Abies chensiensis* Tiegh. | | MH 706706 | 121,795 bp |
| Pinaceae | *Abies* | *Abies chensiensis* Tiegh. | | MH 047653 | 121,498 bp |
| Pinaceae | *Abies* | *Abies delavayi* Franch. | | MH 706709 | 120,141 bp |
| Pinaceae | *Abies* | *Abies delavayi* Franch. | | MK607413 | 120,142 bp |
| Pinaceae | *Abies* | *Abies delavayi* subsp. *fansipanensis*  (Q.P.Xiang, L.K.Fu & Nan Li) Rushforth | | MH 706720 | 120,093 bp |
| Pinaceae | *Abies* | *Abies delavayi* subsp. *fansipanensis*  (Q.P.Xiang, L.K.Fu & Nan Li) Rushforth | | MK607416 | 120,094 bp |
| Pinaceae | *Abies* | *Abies ernestii* Rehd. | | MH 706707 | 121841 bp |
| Pinaceae | *Abies* | *Abies ernestii* var. *salouenensis* (Bordères & Gaussen) W. C. Cheng & L. K. Fu | | MH 706708* | 121,759 bp |
| Pinaceae | *Abies* | *Abies fabri* (Mast.) Craib | | MH 706710 | 120,027 bp |
| Pinaceae | *Abies* | *Abies fanjingshanensis* W. L. Huang, Y. L. Tu & S. Z. Fang | | MH 706717 | 120,057 bp |
| Pinaceae | *Abies* | *Abies fargesii* Franch. | | MH706716 | 121,799 bp |
| Pinaceae | *Abies* | *Abies forrestii* C. C. Rogers | | MH 706715 | 120,022 bp |
| Pinaceae | *Abies* | *Abies georgei* var. *smithii* (Viguie et  Gaussen) Cheng et L | | NC_054152 | 121,191 bp |
| Pinaceae | *Abies* | *Abies kawakamii* (Hayata) T. Ito | | MH 706726 | 121,290 bp |
| Pinaceae | *Abies* | *Abies koreana* E. H. Wilson | | KP 742350 | 121,373 bp |
| Pinaceae | *Abies* | *Abies nephrolepis* (Trautv.) Maxim. | | KT 834974 | 121,336 bp |
| Pinaceae | *Abies* | *Abies nukiangensis* Cheng et L. K. Fu | | MH 706711 | 120,017 bp |
| Pinaceae | *Abies* | *Abies nukiangensis* Cheng et L. K. Fu | | MK607415 | 120,015 bp |
| Pinaceae | *Abies* | *Abies yuanbaoshanensis* Y. J. Lu & L. K. Fu | | MH 706718 | 121,795 bp |
| Pinaceae | *Keteleeria* | *Keteleeria davidiana* (Bertr.) Beissn. | | NC_011930^#^ | 117720 bp |

*: indicated newly generated chloroplast genomes; #：indicated outgroup.

**Table S2.** The chloroplast genome organization of *Abies ernestii* var. *salouenensis*.

| **Gene** | **Position** | **Length (bp)** | **Gene** | **Position** | **Length (bp)** | **Gene** | **Position** | **Length (bp)** |
| --- | --- | --- | --- | --- | --- | --- | --- | --- |
| *psbA* | 1 | 1062 | *psbM* | 33408 | 123 | *rps4* | 69979 | 606 |
| *trnK-UUU* | 1609 | 2596 | *petN* | 34355 | 90 | *trnS-GGA* | 70907 | 87 |
| *matK* | 1855 | 1530 | *trnC-GCA* | 34766 | 71 | *ycf3* | 71731 | 1945 |
| *chlB* | 4918 | 1545 | *rpoB* | 35232 | 3231 | *psaA* | 74279 | 2253 |
| *trnQ-UUG* | 6602 | 72 | *rpoC1* | 38485 | 2761 | *psaB* | 76557 | 2205 |
| *psbK* | 7028 | 180 | *rpoC2* | 41339 | 3657 | *rps14* | 78911 | 300 |
| *psbI* | 7696 | 111 | *rps2* | 45216 | 705 | *trnfM-CAU* | 79338 | 74 |
| *trnS-GCU* | 7882 | 88 | *atpI* | 46224 | 747 | *trnG-UCC* | 79564 | 71 |
| *psaM* | 8175 | 87 | *atpH* | 47659 | 246 | *psbZ* | 79760 | 324 |
| *ycf12* | 8589 | 102 | *atpF* | 48155 | 1337 | *trnS-UGA* | 80323 | 87 |
| *clpP* | 9107 | 591 | *atpA* | 49535 | 1524 | *psbC* | 80614 | 1422 |
| *rps12* | 9871 | 916 | *trnR-UCU* | 51157 | 70 | *psbD* | 81983 | 1062 |
| *rpl20* | 10698 | 351 | *trnG-GCC* | 51433 | 853 | *trnT-GGU* | 84355 | 72 |
| *rps18* | 11328 | 282 | *ycf12* | 52470 | 102 | *trnT-GGU* | 84356 | 71 |
| *rpl33* | 11733 | 207 | *psaM* | 52899 | 87 | *rrn16* | 84787 | 1491 |
| *psaJ* | 12274 | 132 | *trnS-GCU* | 53191 | 88 | *trnI-GAU* | 86651 | 1059 |
| *trnP-UGG* | 12714 | 74 | *psbB* | 53710 | 1527 | *trnA-UGC* | 87783 | 847 |
| *trnW-CCA* | 12956 | 74 | *psbT* | 55311 | 108 | *rrn23* | 88771 | 2807 |
| *petG* | 13158 | 114 | *psbN* | 55500 | 132 | *rrn4.5* | 91682 | 103 |
| *petL* | 13424 | 135 | *psbH* | 55711 | 228 | *rrn5* | 92035 | 121 |
| *psbE* | 14823 | 252 | *petB* | 56096 | 1420 | *trnR-ACG* | 92378 | 74 |
| *psbF* | 15084 | 120 | *petD* | 57730 | 1238 | *trnN-GUU* | 93185 | 72 |
| *psbL* | 15230 | 117 | *rpoA* | 59162 | 1011 | *chlL* | 93536 | 876 |
| *psbJ* | 15465 | 123 | *rps11* | 60232 | 393 | *chlN* | 94528 | 1413 |
| *petA* | 16638 | 960 | *rpl36* | 60707 | 114 | *ycf1* | 96186 | 5880 |
| *cemA* | 17824 | 786 | *infA* | 60923 | 237 | *rps15* | 102393 | 267 |
| *ycf4* | 19050 | 555 | *rps8* | 61303 | 399 | *psaC* | 104337 | 246 |
| *psaI* | 20236 | 111 | *rpl14* | 61847 | 369 | *ccsA* | 105947 | 960 |
| *accD* | 20894 | 969 | *rpl16* | 62332 | 1283 | *trnL-UAG* | 107044 | 78 |
| *trnR-CCG* | 22123 | 74 | *rps3* | 63708 | 654 | *trnP-GGG* | 107274 | 74 |
| *rbcL* | 22441 | 1428 | *rpl22* | 64364 | 402 | *rpl32* | 108375 | 180 |
| *atpB* | 24622 | 1479 | *rps19* | 64805 | 279 | *trnV-GAC* | 109347 | 72 |
| *atpE* | 26109 | 414 | *rpl2* | 65138 | 1500 | *rps7* | 111855 | 468 |
| *trnM-CAU* | 26635 | 73 | *rpl23* | 66658 | 276 | *trnL-CAA* | 113802 | 81 |
| *trnV-UAC* | 26901 | 617 | *trnI-CAU* | 67129 | 74 | *ycf2* | 114614 | 6246 |
| *trnE-UUC* | 31810 | 73 | *trnF-GAA* | 68212 | 73 | *trnH-GUG* | 121146 | 75 |
| *trnY-GUA* | 31950 | 84 | *trnL-UAA* | 68656 | 569 | *trnI-CAU* | 121533 | 74 |
| *trnD-GUC* | 32295 | 74 | *trnT-UGU* | 69642 | 73 |  |  |  |

**
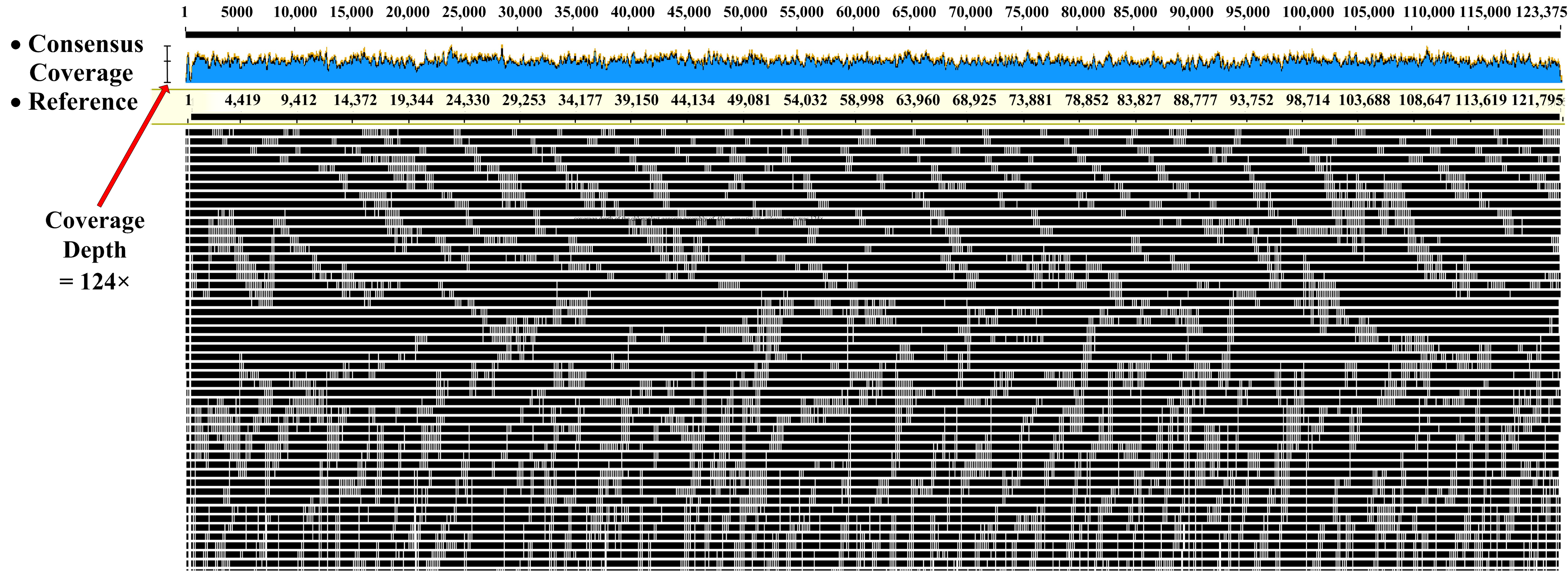
**

**Figure S1**. Overall coverage depth of the chloroplast genome assembly of *Abies ernestii* var. *salouenensis*.

**
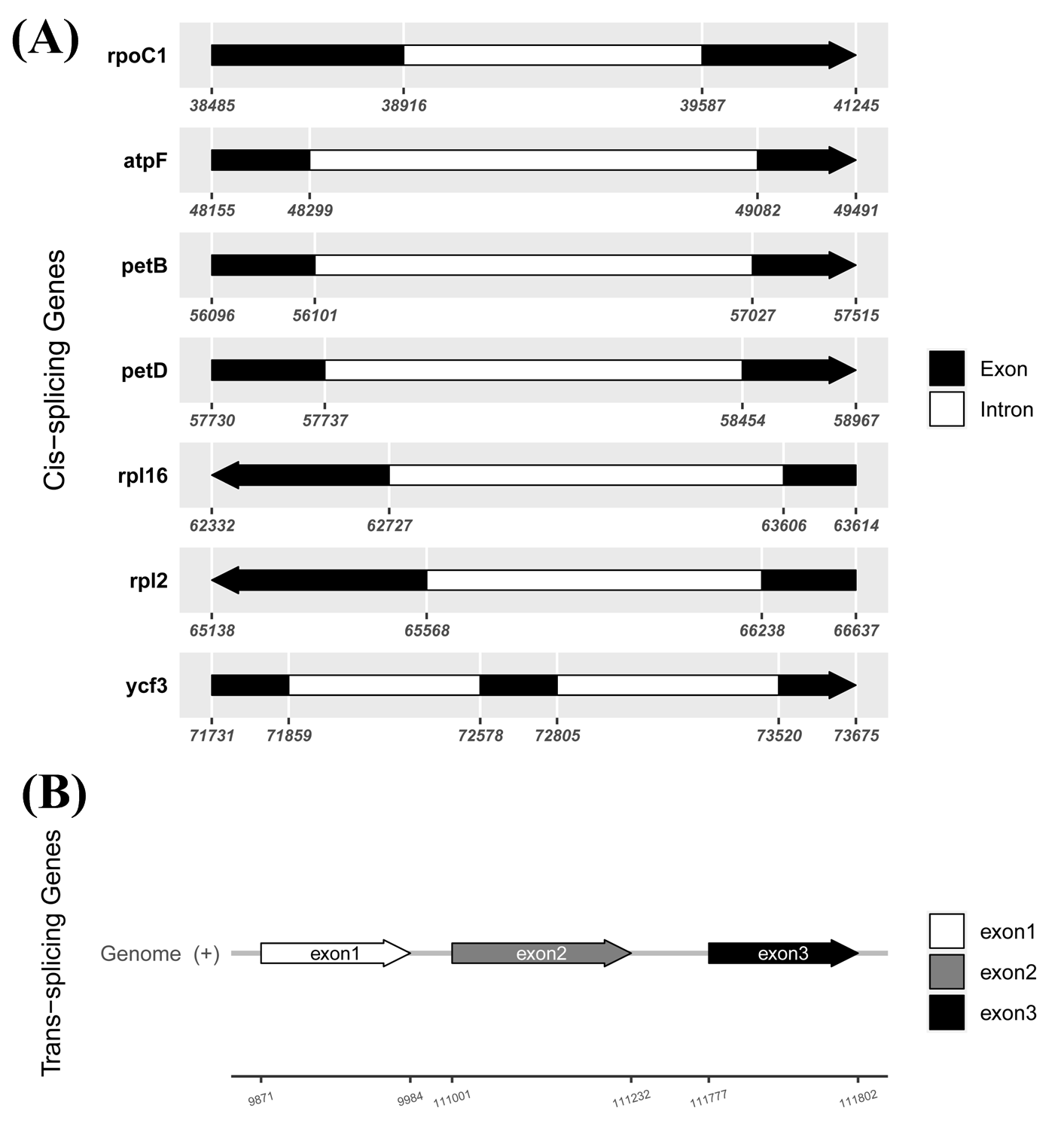
**

**Figure S2**. Schematic map of the cis and trans splicing genes in the chloroplast genome of *Abies ernestii* var. *salouenensis*.

**
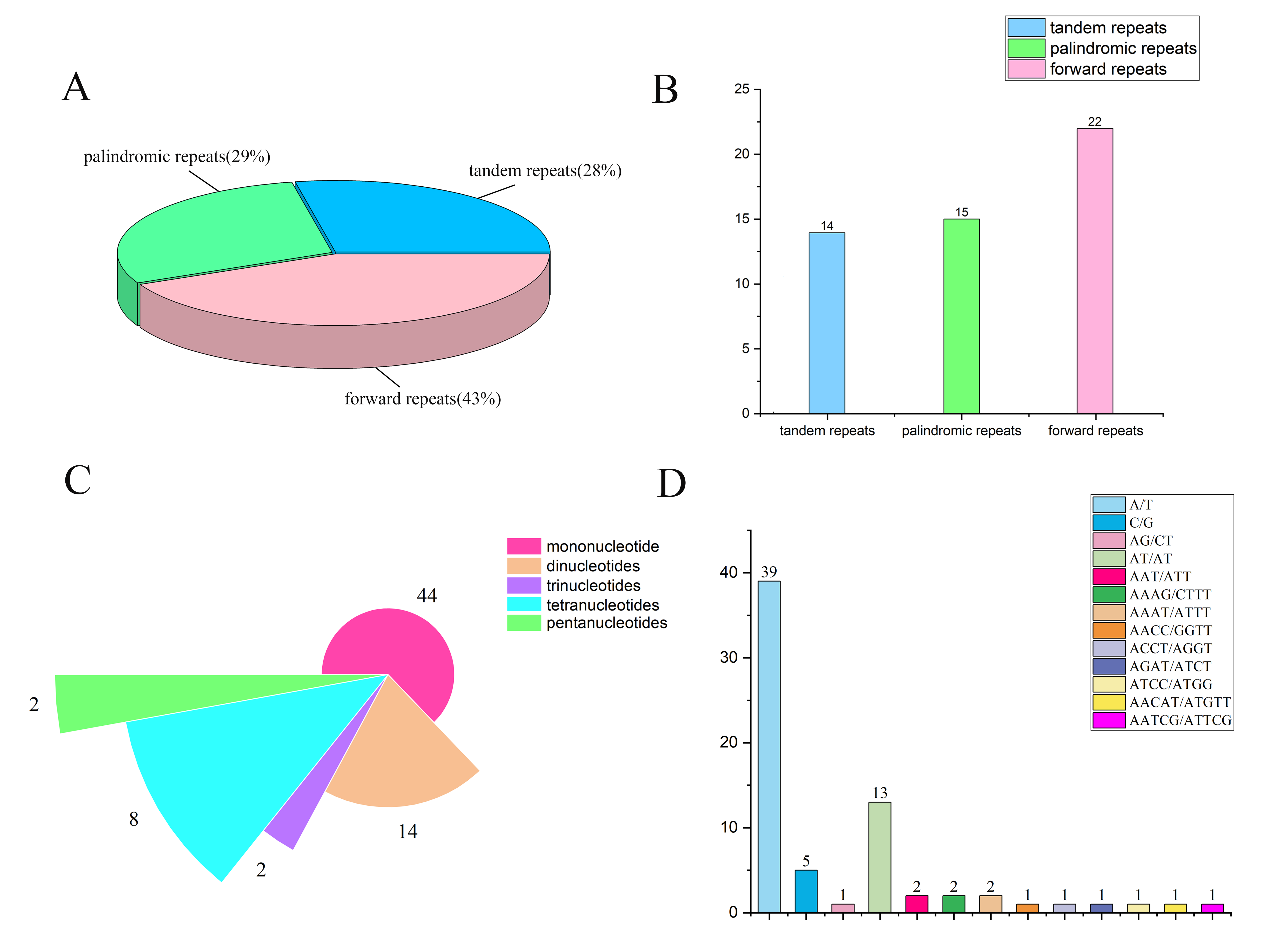
**

**Figure S3**. Types and amount of SSRs (A and B) and long sequence repeats (C and D) in the *Abies ernestii* var. *salouenensis* chloroplast genome.

**
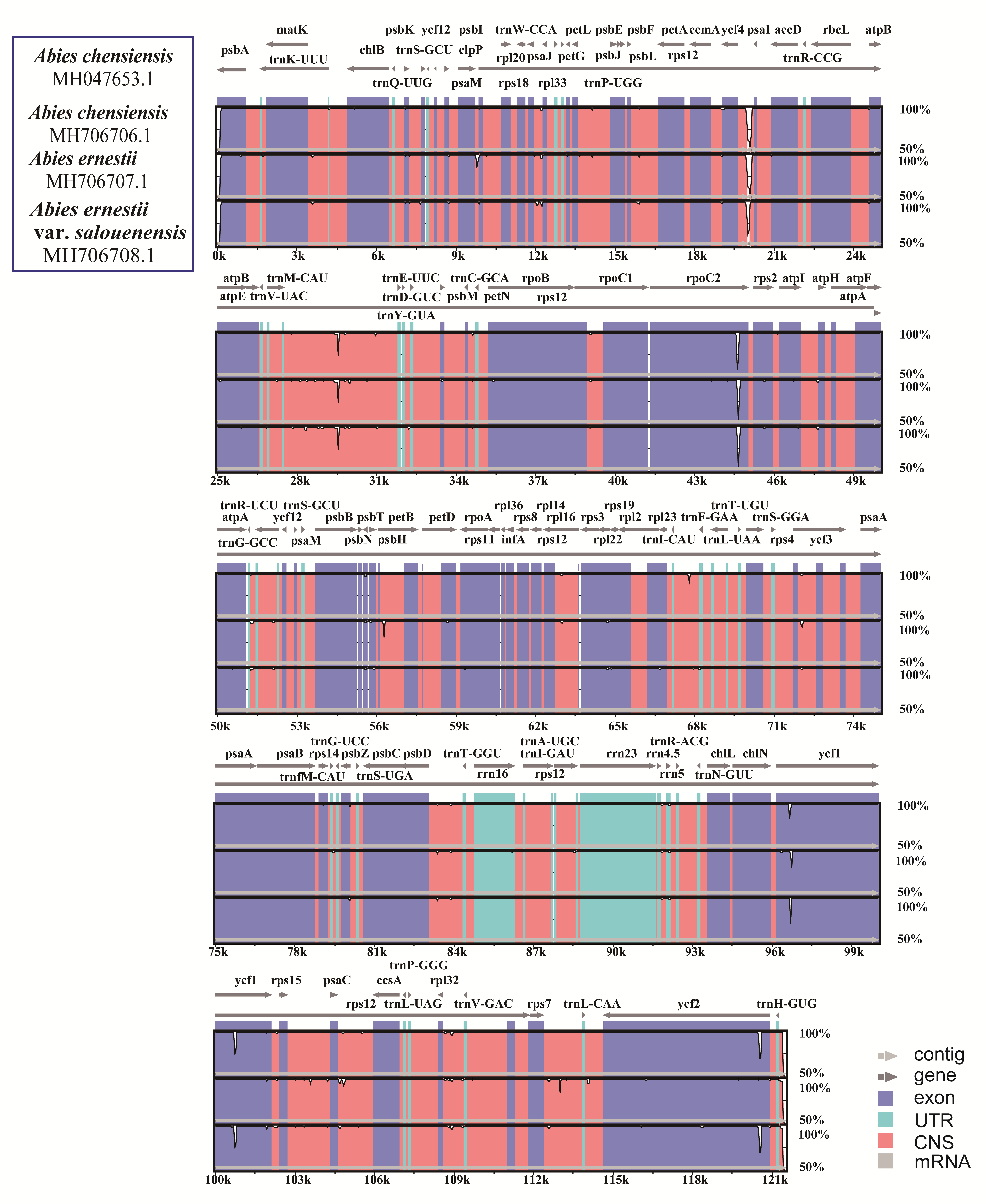
**

**Figure S4**. Comparison of four chloroplast genomes using the mVista alignment program, with *Abies chensiensis* (MH706706) as a reference. The X-axis means the window's midpoint, and the y-axis means nucleotide diversity (Pi). Genome regions are colour-coded as protein-coding, rRNA coding, tRNA coding, or conserved noncoding sequences.
